# One Year Effects of a Workplace Integrated Care Intervention for Workers with Rheumatoid Arthritis: Results of a Randomized Controlled Trial

**DOI:** 10.1007/s10926-016-9639-0

**Published:** 2016-04-07

**Authors:** M. van Vilsteren, C. R. L. Boot, J. W. R. Twisk, R. Steenbeek, A. E. Voskuyl, D. van Schaardenburg, J. R. Anema

**Affiliations:** 1 Department of Public and Occupational Health, EMGO Institute for Health and Care Research, VU University Medical Center, Room BS7-C573, Van der Boechorststraat 7, 1081 BT Amsterdam, The Netherlands; 2 Body@Work, Research Center on Physical Activity, Work, and Health, TNO-VU University Medical Center, Amsterdam, The Netherlands; 3 Department of Health Sciences Section Methodology and Applied Biostatistics, VU University, Amsterdam, The Netherlands; 4 TNO Work, Health and Care, Leiden, The Netherlands; 5 Department of Rheumatology, VU University Medical Center, Amsterdam, The Netherlands; 6 Jan van Breemen Research Institute | Reade, Amsterdam, The Netherlands; 7 Research Center for Insurance Medicine, AMC-UMCG-UWV-VU University Medical Center, Amsterdam, The Netherlands

**Keywords:** Rheumatoid arthritis, Work, Randomized controlled trial, Workplace intervention

## Abstract

*Purpose* To evaluate the effectiveness of a workplace integrated care intervention on at-work productivity loss in workers with rheumatoid arthritis (RA) compared to usual care. *Methods* In this randomized controlled trial, 150 workers with RA were randomized into either the intervention or control group. The intervention group received an integrated care and participatory workplace intervention. Outcome measures were the Work Limitations Questionnaire, Work Instability Scale for RA, pain, fatigue and quality of life (RAND 36). Participants filled out a questionnaire at baseline, and after 6 and 12 months. We performed linear mixed models to analyse the outcomes. *Results* Participants were on average 50 years of age, and mostly female. After 12 months, no significant intervention effect was found on at-work productivity loss. We also found no significant intervention effects on any of the secondary outcomes. *Conclusions* We did not find evidence for the effectiveness of our workplace integrated care intervention after 12 months of follow up. Future studies should focus on investigating the intervention in groups of workers with severe limitations in work functioning, and an unstable work situation.

## Introduction

Rheumatoid arthritis (RA) is a chronic autoimmune disease which is characterized by inflamed joints. Despite treatment of RA with conventional disease modifying anti-rheumatic drugs, or more recently, with biological therapeutics, RA still leads to profound symptoms [1]. These symptoms, such as stiffness of the joints, pain and fatigue, might fluctuate.

RA also impacts a person’s working life [2]. Many studies have shown that permanent work disability occurs more frequently in patients with RA when compared to the general population, although there is a slight decrease in work disability rates during recent years [3–6]. For example in the study of Sokka et al. [3], it was shown that the probabilities for continuing work were 80 % after 2 years, and 68 % after 5 years for workers with RA. Before a patient becomes permanently work disabled, he goes through a process in which continuing work becomes more and more difficult. At first, patients might experience at-work productivity loss. This means that a patient is still present at work, but is limited in meeting work demands, and is therefore less productive at work. If work functioning becomes very difficult, a patient might call in sick. Sick leave can lead to permanent work disability, or patients can return to work. The longer the period of sick leave, the harder it is to return to work [7]. For this reason, to prevent patients with RA becoming permanently work disabled, interventions should focus on patients in an early stage, preferably patients who are not yet sick-listed.

Patients with RA experience reduced health related quality of life compared to the general population [8], which might be even more reduced in case of work disability. Previous studies showed that reduced work capacity not only leads to financial restrains, but is also related to reduced quality of life [9, 10]. Patients with RA who are restricted in work might furthermore experience feelings of hopelessness, sadness, anger and irritation [11].

In order to support workers with RA in maintaining and improving productivity at work, the Care for Work project was initiated. In this project, an intervention program, consisting of integrated care and a participatory workplace intervention, was evaluated in a group of workers with RA.

The aim of this study was to evaluate the effectiveness of the intervention program on at-work productivity loss, pain, fatigue and quality of life compared to usual care, after 12 months of follow-up. We hypothesize that the intervention program leads to a reduction in at-work productivity loss, improved quality of life, and less pain and fatigue.

## Materials and Methods

### Design

The effectiveness of the Care for Work intervention program was evaluated using a randomized controlled trial (RCT). Participants who gave written informed consent took part in three measurements. One before the start of the intervention, baseline (T0), after 6 (T1) and after 12 months (T2). The study design and procedures were approved by the Medical Ethics Committee of the Slotervaart hospital and Reade, and the Medical Ethics Committee of the VU University Medical Center. This trial was registered in the Dutch Trial Register (NTR2886). Details of the trial have been described elsewhere [12].

### Participants

Participants were recruited at Reade, Amsterdam, the outposts of Reade, and the department of rheumatology of the VU University Medical Center, Amsterdam. Eligible patients were 18–64 years of age, diagnosed with RA, had a paid job for at least 8 h per week (employment contract or self-employed), and experienced at least minor difficulties in functioning at work. Patients could not participate in case of severe comorbidity, inability to read or understand Dutch language, and in case of more than 3 months of sick leave duration at time of inclusion. Eligible patients received an information letter about the project from their own rheumatologist.

### Randomization and Blinding

After baseline measurements, participants were individually randomized into either the intervention group or control group (usual care). Participants were pre-stratified by three prognostic factors; sex, number of work hours per week (<20 h and >20 h per week), and whether a participant performed heavy or light physically/mentally demanding work, based on the classification of De Zwart [13]. Randomization occurred with the minimization method, by applying a software program called Minim [14], which allows pre-stratification by several prognostic factors even in small samples [15, 16]. Due to the character of the intervention, participants, therapists and researchers could not be blinded for the allocated treatment.

### Intervention

All patients received usual rheumatologist-led care. The patients in the intervention program also received the Care for Work intervention program [12]. The program consisted of two components which complemented each other; integrated care and a participatory workplace intervention. Integrated care was delivered by a multidisciplinary team, which consisted of a trained clinical occupational physician (who acted as care manager), a trained occupational therapist, and the patients’ own rheumatologist. The care manager coordinated care and communicated with members of the multidisciplinary team, the patient’s supervisor, occupational physician and general practitioner. The care manager performed the intake of the patient in the intervention, which consisted of history taking and physical examination to identify functional limitations at work and factors that could influence functioning at work. The care manager proposed a treatment plan at the end of the first consultation. After the patient’s consent, the care manager sent the treatment plan to the other members of the multidisciplinary team. The patients visited the care manager again after 6 and 12 weeks to evaluate. After the occupational therapist received the treatment plan from the care manager, the occupational therapist started the participatory workplace intervention, which is based on active participation and strong commitment of both the patient and supervisor. The workplace intervention was based on participatory ergonomics [17–19]. The aim of the workplace intervention was to achieve consensus between patient and supervisor regarding feasible solutions for obstacles for functioning at work. After consensus, the occupational therapist, patient and supervisor agreed on a plan of action. The patient and supervisor were responsible for implementing the plan of action. The occupational therapist evaluated implementation of the action plan after 4 weeks.

### Measurements

#### Primary Outcome

At-work productivity loss was operationalized as hours lost from work due to presenteeism. Presenteeism refers to being present at work, but being limited in meeting work demands, and hence, at-work productivity is reduced. We measured at-work productivity loss with the Work Limitations Questionnaire (WLQ). A score was calculated based on 25 items which presents the percentage of at-work productivity loss. This score was multiplied by the number of working hours per 2 weeks, resulting in an estimation of the hours of experienced at-work productivity loss during the past 2 weeks. The WLQ consists of four subscales (time management demands, physical demands, mental-interpersonal demands, and output demands) which are calculated into scores ranging from 0 (no limitations) to 100 (highest limitations). The internal reliability is high for the separate WLQ subscales [20]. The good validity and reliability of the WLQ concerning RA have been shown in several previous studies [20–22].

#### Secondary Outcomes

##### Quality of Life

We measured quality of life with the RAND 36 [23, 24]. The RAND 36 consists of nine subscales, we included four subscales in our analyses. These subscales are mental health, physical role limitations, physical functioning, and perceived health change. The subscales of the RAND 36 are transformed into a scale score ranging from 0 to 100. A higher score indicates better health.

##### Pain and Fatigue

Pain and fatigue were measured with single items using visual analogue scales (VAS) [25, 26]. Studies have shown that a single item VAS for fatigue and pain performs as well as or better than longer scales in respect to sensitivity to change [26, 27]. We asked patients to indicate their perceived pain/fatigue today. VAS scales ranged from 0 to 10, with 0 meaning no pain/fatigue at all, and 10 meaning a lot of pain/very tired.

##### Work Instability

Work instability was measured with the RA Work Instability Scale (RA WIS) [28, 29]. The RA WIS contains 23 statements such as ‘I’m getting up earlier because of the arthritis’. By counting the statements answered by yes, the RA WIS score is calculated, leading to a score between 0 and 23. A higher score indicates more work instability.

### Potential Confounders

At baseline, data on potential confounders were collected. We collected age and gender from patient medical records. Education level was measured using a single item in the questionnaire. Low education was operationalized as primary school, middle education or basic vocational education. Middle education was operationalized as secondary vocational education or intermediate vocational education. High education was operationalized as higher vocational education or a university degree. Whether comorbidity was present (yes/no) was assessed with a list of 15 common comorbidities. The Disease Activity Score of 28 joints (DAS28) was assessed as a part of usual care and was collected from patient records. The DAS28 score was based on the number of tender and swollen joints in 28 joints, the erythrocyte sedimentation rate (ESR) and the patient’s general health measured on a VAS of 100 mm [30]. We furthermore retrieved the prescription of biological therapeutics from the patient medical records. Disease duration was investigated by one open-ended question about the year of the RA diagnosis. Daily functioning was measured with the Health Assessment Questionnaire (HAQ), a reliable and valid questionnaire [31]. We asked participants whether they were satisfied with their job (not/moderately satisfied or (very) satisfied). We measured co-worker support, supervisor support, decision authority, physical job demands and psychological job demands with the Job Content Questionnaire (JCQ) [32]. We furthermore included baseline data of all of the primary and secondary outcomes described above as potential confounders.

Co-interventions were investigated by one item in the questionnaire; we asked participants whether their work situation was adapted during the past 6 months, independent of the Care for Work project.

### Sample Size

The sample size was calculated based on the number of participants needed to identify an effect on at-work productivity loss, which was measured with the WLQ. We assumed that a difference of 2 h per 2 weeks was a relevant difference. This is based on a study where an average of four lost hours per 2 weeks (SD: 3.9) was found with the WLQ [33]. A 2 h per 2 weeks difference implies a moderate standardized effect of 0.5. Power analysis revealed a sample size of 71 patients per group. Assuming a dropout rate of 15 %, 142 patients had to be included in total, with a power of 0.80 and an alpha of 0.05.

### Statistical Analyses

We performed linear mixed models with each outcome measure as dependent variable. Intervention or control group was the independent variable and all analyses were adjusted for the baseline value of the outcome. Time of the follow-up measurements was the fixed factor (T1: 6-months follow-up, T2: 12-months follow-up). Data were analyzed according to the intention-to-treat principle, indicating that all participants were analyzed according to the condition they were allocated to, despite whether they had engaged in the intervention. We performed crude and adjusted analyses. To select potential confounders, we checked baseline differences between the intervention and control group, and selected those variables with a *p* value <0.4. Secondly, we assessed correlations between the remaining covariates and the outcome. If Pearson’s R was higher than 0.7, the covariate with the weakest correlation with the outcome was not included as confounder. The remaining covariates were entered into the adjusted model. We checked effect modification by co-interventions by adding an interaction term to the adjusted model. *p* values <0.05 were considered statistically significant. All analyses were performed using SPSS software (version 20.0).

## Results

**Fig. 1:**
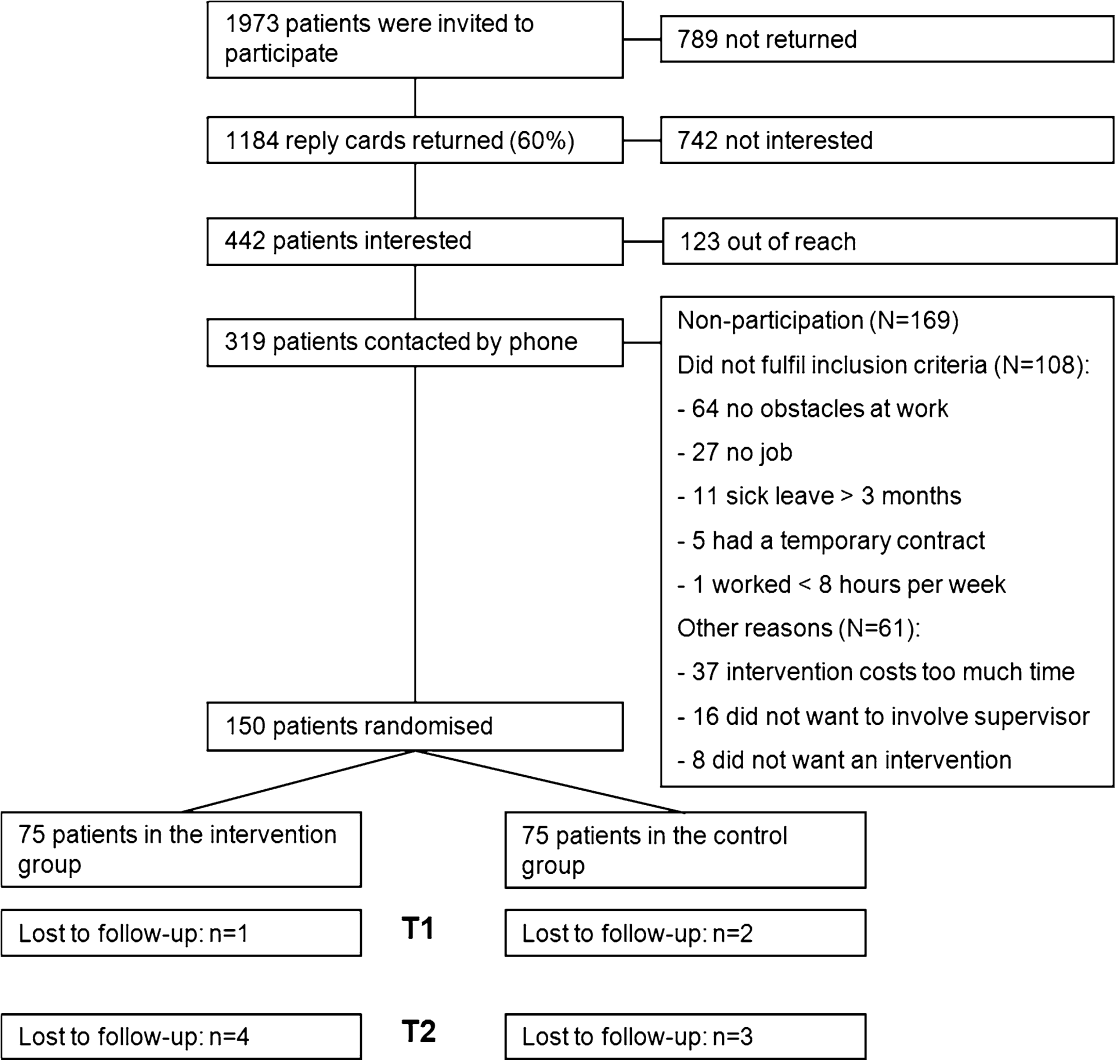
Flow diagram Care for Work study

In total, 1973 RA patients were invited to participate (Fig. 1). We did not have information about work status of the patients that were invited by the rheumatologist. In total, 1531 patients did not return the reply card or indicated that they were not interested, possibly because they did not have a paid job. Eventually, 150 patients completed the baseline questionnaire and were randomized into either the control or intervention group. After 6 months, 147 participants completed the first follow-up questionnaire, and after 12 months, 143 participants completed the second follow-up questionnaire. Loss to follow-up was therefore 4.7 %.

Table 1 presents the baseline characteristics of the study population. Participants were on average 50 years of age, and mostly female. Participants scored 0.8 on the HAQ, and the DAS28 was on average 2.7. Almost half of our study population had ever used biological therapeutics (45 % in the control group and 48 % in the intervention group).

**Table 1:** Baseline characteristics of the study population n = 150

Variable		Control n = 75	Intervention n = 75
Gender^a^	Male	12 (16 %)	12 (16 %)
	Female	63 (84 %)	63 (84 %)
Comorbidity present^a^	No	24 (32 %)	29 (39 %)
	Yes	51 (68 %)	46 (61 %)
Education^a^	Low	16 (21 %)	16 (21 %)
	Middle	26 (35 %)	22 (29 %)
	High	33 (44 %)	37 (49 %)
Job satisfaction^a^	Satisfied	57 (76 %)	46 (61 %)
	Not satisfied	18 (24 %)	29 (39 %)
Age^b^	Years	49.6 (8.7)	49.8 (8.6)
HAQ^b^	0–3	0.8 (0.5)	0.8 (0.6)
DAS28^b^	0–	2.7 (1.2)	2.7 (1.3)
Duration since diagnosis^b^	Years	10.0 (8.6)	10.9 (9.1)
Biological use^a^	No	40 (53 %)	38 (51 %)
	Yes	34 (45 %)	36 (48 %)
WLQ lost hours^b^	Hours	3.4 (2.8)*	4.6 (2.5)*
RAWIS^b^	0–23	7.9 (4.8)*	9.8 (4.6)*
RA WIS corresponding risk	Low	44 (59 %)	37 (49 %)
	Medium	27 (36 %)	29 (39 %)
	High	4 (5 %)	9 (12 %)
RAND physical functioning^b^	0–100	65.7 (21.0)	68.5 (22.0)
RAND physical role limitations^b^	0–100	53.7 (40.4)	42.3 (39.6)
RAND mental health^b^	0–100	80.1 (14.4)*	74.4 (14.2)*
RAND perceived health change^b^	0–100	51.7 (24.1)	51.7 (29.2)
Co-worker support JCQ^b^	1–4	3.1 (0.5)	3.1 (0.5)
Supervisor support JCQ^b^	1–4	3.0 (0.6)	3.0 (0.6)
Decision authority JCQ^b^	1–4	2.7 (0.5)	2.8 (0.6)
Psychological job demands JCQ^b^	1–4	2.7 (0.3)	2.6 (0.3)
Physical job demands JCQ^b^	1–4	2.0 (0.6)	2.0 (0.6)

* Significant difference *p* < 0.05

^a^n (%)

^b^m (SD)

Due to a systematic error in our minimisation procedure, a subgroup of 37 participants was considered at risk to be mistakenly allocated to the control or intervention group. For this reason we conducted a sensitivity analysis on the subgroup in which we left out the 37 participants at risk, to determine the impact of the potential bias on the study results. In this subgroup, 55 patients were randomised into the intervention group and 58 patients into the control group. Before conducting the sensitivity analyses, a change in the regression coefficients of >10 % between the two analyses was defined as a relevant difference. All analyses were replicated by an independent researcher.

### Intervention Effects

#### Primary Outcome

At baseline, the intervention group lost on average 4.6 h per 2 weeks due to at-work productivity loss. The control group lost 3.4 h per 2 weeks. After 12 months, the intervention group remained constant, while the control group increased slightly over time with 0.1 h per 2 weeks. No significant intervention effects were observed on at-work productivity loss (the difference between the two groups on average over time (B) was 0.24 (95 % CI −0.43 to 0.90). We also did not find intervention effects on any of the four subscales of the WLQ. No increase or decrease was observed over time in any of the two groups (Table 2). The use of co-interventions was not found to be a relevant effect modifier in the analyses.

**Table 2:** Intervention effects after 12 months of follow-up

Outcomes	Group (N = 150)	Baseline mean (SD)	T1 mean (SD)	T2 mean (SD)	B (95 % CI) crude^a^	B (95 % CI) adjusted^b^
At-work productivity loss	Intervention	4.6 (2.5)	4.5 (3.1)	4.6 (3.1)	0.24 (−0.43; 0.90)	0.25 (−0.31; 0.80)
	Control	3.4 (2.8)	3.7 (2.5)	3.5 (2.1)		
Time management demands (WLQ)	Intervention	34.8 (24.3)	35.8 (20.4)	35.6 (19.0)	3.82 (−1.43; 9.08)	2.75 (−1.48; 6.99)
	Control	29.5 (23.0)	28.6 (18.9)	29.4 (22.8)		
Physical demands (WLQ)	Intervention	31.0 (20.9)	27.1 (20.2)	31.4 (19.2)	−1.48 (−6.60; 3.63)	−0.86 (−5.54; 3.82)
	Control	26.9 (19.9)	30.0 (21.7)	27.1 (20.7)		
Mental-interpersonal demands (WLQ)	Intervention	24.1 (18.8)	23.2 (19.0)	24.1 (20.4)	1.58 (−2.82; 5.98)	0.78 (−2.85; 4.41)
	Control	19.0 (18.4)	19.3 (13.7)	19.1 (17.1)		
Output demands (WLQ)	Intervention	32.1 (19.6)	29.0 (19.6)	28.9 (19.0)	0.84 (−4.05; 5.74)	0.68 (−3.36; 4.73)
	Control	22.2 (18.7)	22.1 (17.4)	22.8 (18.9)		
Work instability	Intervention	9.8 (4.6)	8.6 (4.6)	9.3 (5.2)	−0.29 (−1.41; 0.84)	0.10 (−0.84; 1.05)
	Control	7.9 (4.8)	7.9 (5.9)	7.7 (6.0)		
Pain	Intervention	3.7 (2.5)	3.8 (2.6)	4.1 (2.6)	0.19 (−0.41; 0.78)	0.51 (−0.003; 1.02)
	Control	3.7 (2.5)	3.8 (2.4)	3.9 (2.4)		
Fatigue	Intervention	4.8 (2.5)	5.1 (2.7)	5.6 (2.7)	0.27 (−0.36; 0.90)	0.49 (−0.07; 1.05)
	Control	4.4 (2.6)	4.7 (2.7)	5.1 (2.7)		
Physical role limitations	Intervention	42.3 (39.6)	48.0 (40.5)	44.0 (41.7)	−3.29 (−13.92; 7.34)	−0.29 (−9.02; 8.44)
	Control	53.7 (40.4)	52.1 (42.8)	56.0 (40.2)		
Physical functioning	Intervention	68.5 (22.0)	70.7 (20.4)	66.0 (20.9)	−1.69 (−5.91; 2.53)	−2.50 (−5.91; 0.91)
	Control	65.7 (21.0)	68.7 (19.4)	69.3 (20.0)		
Mental health	Intervention	74.3 (14.2)	78.4 (14.0)	75.4 (15.8)	0.78 (−2.91; 4.46)	0.73 (−2.30; 3.78)
	Control	80.1 (14.4)	79.8 (15.7)	79.6 (15.7)		
Perceived health change	Intervention	51.7 (29.2)	53.0 (30.1)	52.8 (23.4)	4.89 (−1.66; 11.44)	3.68 (−2.38; 9.74)
	Control	51.7 (24.1)	49.0 (22.6)	46.5 (24.2)		

*B* difference between the groups on average over time, *CI* confidence interval

^a^Adjusted for baseline value of the outcome

^b^Further adjusted for at-work productivity loss, work instability, decision authority, psychological job demands, physical functioning, physical role limitations, mental health, fatigue, job satisfaction, and comorbidity

#### Secondary Outcomes

Table 2 shows the results of the mixed model analyses on the secondary outcomes. We found no significant intervention effects on the secondary outcomes. For work instability, both groups remained fairly constant over time, leading to a B of −0.29 (95 % CI −1.41 to 0.84). The analyses on pain and fatigue did not show a considerable effect of the intervention [B 0.19 (95 % CI −0.41 to 0.78) and B 0.27 (95 % CI −0.36 to 0.90), respectively]. Both groups showed a slight increase in pain and fatigue over time. On physical role limitations of the RAND36, both groups improved slightly, but the difference between the groups was not significant [B −3.29 (95 % CI −13.92 to 7.34)]. We found no significant intervention effects on physical functioning and mental health [B −1.69 (95 % CI −5.91 to 2.53) and B 0.78 (95 % CI −2.91 to 4.46) respectively]. The intervention group improved in perceived health change, while the control group worsened, although this difference was also not significant [B 4.89 (95 % CI −1.66 to 11.44)].

### Sensitivity Analysis

In the subgroup, we found no statistically significant effect of the intervention on at-work productivity loss [B 0.39 (95 % CI −0.24 to 1.01)].

## Discussion

### Main Findings

We evaluated an intervention program consisting of integrated care and a participatory workplace intervention and investigated its effectiveness after 12 months of follow-up. We found no intervention effects on any outcome: at-work productivity loss, work instability, pain, fatigue, and quality of life.

### Comparison with Other Studies

The intervention we evaluated has been proven effective in a previous study. Lambeek et al. evaluated the same intervention for workers with low back pain who were on sick leave. The median time until sustainable return to work was 88 days in the intervention group compared to 208 days in the usual care group (*p* = 0.003) [34]. The differences between our study and the study of Lambeek et al. are the study population and the outcome. We did not include workers who were on sick leave, and focused on functioning at work instead of return to work. In a situation where a worker is on sick leave, the need to act is much higher than was the case in our study, resulting in more room for improvement. Workers in our study were present at work, and conducted their normal tasks. This lowers the necessity for both the worker and supervisor to discuss barriers at the workplace, resulting in improvements at the workplace.

If we compare our study to other studies for workers with RA with a focus on work functioning and sick leave, we come across either medical, or work-related interventions. An example of a medical study is performed by Eriksson et al. [35], who analysed the effect of biological treatment on sick leave. It was shown that the group treated with a biological had better radiological outcomes, but this result did not translate into better work outcomes. This suggests that a purely medical intervention alone is not enough to improve work participation.

An example of a work-related intervention is described in a Dutch study in which a multidisciplinary job-retention vocational rehabilitation program was compared to usual care [36]. The intervention aimed to guide patients and adapt an intervention to the specific needs of a patient. At follow-up, no differences were found between the groups on job retention. An important difference with our intervention is that this intervention did not incorporate active involvement of the workplace (i.e. a work visit, or involvement of the supervisor). Macedo et al. [37] evaluated an intervention which, a work visit was included, besides other intervention components. Patients were included if they had a medium or high work disability risk according to the RA WIS. The intervention was significantly beneficial on work instability, work satisfaction, and work performance. This strengthens our hypothesis that the workplace should be included in the intervention to enhance work-related outcomes in RA patients.

When comparing our study population with the study population of the Macedo study, they are comparable in age, disease duration, and the fact that mostly women participated. The big difference however, is the score of the study populations on work instability measured with the RA WIS. The RA WIS leads to a score between 0 and 23 which indicates the disability risk. A higher score indicates a higher risk. In our study, participants scored on average between eight and nine points on the RA WIS, which indicates low risk. If we look at the corresponding risk, approximately 36–39 % of our participants had a medium risk at baseline, and between 5 and 12 % a high risk of work disability. As we drew our study sample from the general RA population of the participating hospitals, this sample might have been too stable in its work situation and too little limited in work functioning. Participants in the Macedo study had either medium risk (a score between 10 and 17 points) or high risk (a score >17 points) at baseline. The findings of the Macedo study indicate that participants with a higher risk for work disability might have more to gain from a work-related intervention. This was also emphasized in the study of Baldwin et al. [38]. They evaluated a workplace ergonomic intervention carried out at the workplace. Although they found a significant difference between groups, i.e. the intervention group reported less arthritis-related impact on their work. In addition, they included patients with a mild degree of limitations in work functioning at baseline, which might explain the small changes over time they found [38].

### Strengths and Limitations

We measured at-work productivity loss with the WLQ. This is a strength because of the reliability and validity of the WLQ among workers with RA. However, up till now, there is no consensus about which questionnaire to use in order to measure at-work productivity loss. As was shown by Zhang et al. [33], estimates of at-work productivity loss vary greatly according to the instrument chosen. In the Zhang study, 250 workers with either RA or osteoarthritis were asked to fill out a questionnaire containing four instruments to measure at-work productivity loss [WLQ, Health and Labour Questionnaire (HLQ), the World Health Organization’s Health and Work Performance Questionnaire (HPQ), and the Work Productivity and Activity Impairment Questionnaire (WPAI)]. The average number of lost hours per 2 weeks ranged from 1.6 to 14.2 h. This highlights the variety among instruments to measure at-work productivity loss. The use of another instrument might therefore have yielded a different magnitude of at-work productivity loss. However, as we focussed on group differences over time, we do not expect differences in the effects reported in this study.

A study weakness is the potential bias due to the systematic error in our minimisation procedure, which led to a difference in the effect estimate of more than 10 % between analyses on the total group and subgroup. However, our sensitivity analysis did not lead to a different conclusion, as no effects were present in any analysis. Based on these findings the allocation error did not influence our conclusions about the effectiveness of our intervention program.

A study weakness is the relatively mild degree of limitations in work functioning (low work instability at baseline, and little at-work productivity loss). Such low scores suggest a decreased opportunity to improve work functioning and detect changes. The results are also limited because our study population consisted of workers with a mean disease duration of 10 years (which implies a stable work situation), and the fact that workers could indicate themselves whether they wanted to participate. This suggests that workers who did participate, were under the impression that their supervisor would be supportive of the intervention [39].

### Study Implications

We were not able to find evidence for the effectiveness of a workplace integrated care intervention for workers with RA. Nevertheless, workers that are limited in their work functioning are a relevant target group for future interventions as sustained employability is an important goal for society. It is important to support this group in order to keep them at work, and prevent future work disability. The target group of a workplace integrated care intervention should be critically addressed in future studies. Our study sample consisted of RA patients between 18 and 64 years with paid work and at least minor limitations at work. For future studies, we propose to focus on those workers who are severely limited in their work functioning, and whose work situation is unstable (i.e. early in the disease course). We furthermore propose to conduct studies with a longer follow-up duration.

It is furthermore important to proceed with research on which method is most accurate in measuring at-work productivity loss. As estimates from different measurement instruments can vary, consensus concerning how to measure at-work productivity loss should lead to better comparability between studies, and better insight into the magnitude of at-work productivity loss.

## Conclusions

The workplace integrated care intervention evaluated in this study, did not show any effect on the predefined outcomes at-work productivity, work instability, pain, fatigue and quality of life. Future research should focus on evaluating the intervention in groups of workers with severe limitations in work functioning and an unstable work situation.
